# Lessons on Learning in Later Life With the MIT AgeLab 85+ Lifestyle Leaders

**DOI:** 10.1093/geroni/igab046.722

**Published:** 2021-12-17

**Authors:** Taylor Patskanick, Julie Miller, Lucas Yoquinto, Lisa D'Ambrosio, Joseph Coughlin

**Affiliations:** 1 MIT, Somerville, Massachusetts, United States; 2 MIT, Cambridge, Massachusetts, United States; 3 Massachusetts Institute of Technology, Cambridge, Massachusetts, United States

## Abstract

Previous research has established the role of lifelong learning in promoting psychological wellbeing and active aging. Population aging necessitates an understanding of the unique opportunities and challenges around formal and informal learning in later life. This paper will share findings from a mixed methods study with the MIT AgeLab 85+ Lifestyle Leaders, a panel comprised of octogenarians and nonagenarians from across the United States. Drawing on an online survey and virtual focus groups with 29 Lifestyle Leaders from January 2021, findings suggest the Lifestyle Leaders most often learned new things from talking with others (46%) and reading print (54%) or online (54%) sources. The majority were familiar with attending in-person lectures or classes (89.7%) and were now using videoconferencing to do these (78.6%). A majority (56.7%) had or are currently participating in a lifelong learning program. Most consider themselves lifelong learners and described this around remaining curious and engaged with life, choices around what one learns, and greater enjoyment of learning. In the survey, a plurality of Lifestyle Leaders indicated the top two challenges affecting their ability to learn were sensory burdens (e.g., hearing loss, declining eyesight) (35%) and their energy level (32.4%); focus group data revealed that recall also is a barrier. Focus group data further highlighted generational experiences around early life learning and career paths, specifically how gender roles, diagnoses of learning disabilities, and evolving digital technology have affected these and changed over the course of their lifetimes.

